# On the optimization of low-cost FDM 3D printers for accurate replication of patient-specific abdominal aortic aneurysm geometry

**DOI:** 10.1186/s41205-017-0023-2

**Published:** 2018-01-17

**Authors:** Michael Chung, Norbert Radacsi, Colin Robert, Edward D. McCarthy, Anthony Callanan, Noel Conlisk, Peter R. Hoskins, Vasileios Koutsos

**Affiliations:** 1 The School of Engineering, Institute for Materials and Processes, The University of Edinburgh, Robert Stevenson Road, Edinburgh, EH9 3FB UK; 2 The School of Engineering, Institute for Bioengineering, The University of Edinburgh, Max Born Crescent, Edinburgh, EH9 3FB UK; 3 Centre for Cardiovascular Sciences, The University of Edinburgh, 47 Little France Crescent, Edinburgh, EH16 4TJ UK

**Keywords:** 3D printing, Abdominal aortic aneurysms, Rapid prototype, Flexible, Transparent, Accurate, Thermoplastic polyurethane

## Abstract

**Background:**

There is a potential for direct model manufacturing of abdominal aortic aneurysm (AAA) using 3D printing technique for generating flexible semi-transparent prototypes. A patient-specific AAA model was manufactured using fused deposition modelling (FDM) 3D printing technology. A flexible, semi-transparent thermoplastic polyurethane (TPU), called Cheetah Water (produced by Ninjatek, USA), was used as the flexible, transparent material for model manufacture with a hydrophilic support structure 3D printed with polyvinyl alcohol (PVA). Printing parameters were investigated to evaluate their effect on 3D–printing precision and transparency of the final model. ISO standard tear resistance tests were carried out on Ninjatek Cheetah specimens for a comparison of tear strength with silicone rubbers.

**Results:**

It was found that an increase in printing speed decreased printing accuracy, whilst using an infill percentage of 100% and printing nozzle temperature of 255 °C produced the most transparent results. The model had fair transparency, allowing external inspection of model inserts such as stent grafts, and good flexibility with an overall discrepancy between CAD and physical model average wall thicknesses of 0.05 mm (2.5% thicker than the CAD model). The tear resistance test found Ninjatek Cheetah TPU to have an average tear resistance of 83 kN/m, higher than any of the silicone rubbers used in previous AAA model manufacture. The model had lower cost (4.50 GBP per model), shorter manufacturing time (25 h 3 min) and an acceptable level of accuracy (2.61% error) compared to other methods.

**Conclusions:**

It was concluded that the model would be of use in endovascular aneurysm repair planning and education, particularly for practicing placement of hooked or barbed stents, due to the model’s balance of flexibility, transparency, robustness and cost-effectiveness.

**Electronic supplementary material:**

The online version of this article (10.1186/s41205-017-0023-2) contains supplementary material, which is available to authorized users.

## Background

An abdominal aortic aneurysm (AAA) involves the weakening and enlargement of the lower part of the aorta, due to the degradation of elastin in the arterial wall [1–3]. Rupture of the AAA has a fatality rate of 90% [4]. If diagnosed before rupture, patients with AAA are evaluated for elective surgical repair. Current clinical practice for rupture risk assessment is based on measurement of the diameter of the largest part of the aneurysm [2, 5]. In case the diameter exceeds a threshold value (5.5 cm for men, 5.0 cm for women), patients are considered for repair. If the diameter is less than the threshold patients are put on a screening program. Traditional AAA repair involves open surgery in which the aneurysm is surgically exposed and replaced with a graft which is connected to the aorta. Increasingly, AAA repair is performed using a less invasive procedure involving arterial puncture and the deployment of the graft by catheter. This is referred to as ‘endovascular aneurysm repair’ or EVAR [6]. Surgical repair by EVAR has a number of potential complications including migration of the graft and endoleaks (pooling of blood outside the graft within the excluded aneurysmal sac). There are issues concerning surgical training in EVAR and in surgical planning for the individual patient. Approaches to surgical training and planning for EVAR include the use of virtual reality [7, 8] and experimental systems based on realistic models of AAA [9, 10]. It is the experimental systems which are of interest in the current paper and are further considered below.

Physical models of aneurysms (both abdominal aortic and cerebral) have been manufactured for a variety of scientific purposes; inclusion in experimental flow systems to study flow patterns and pressures [11, 12] for validation of rupture site prediction made using finite element analysis [13], for laboratory investigations of migration of stent grafts [14], and in experimental systems for simulation of interventional procedures [9, 10, 15]. Manufacturing techniques may be broadly divided between casting techniques and 3D printing. Casting of aneurysm phantoms [11–17] is usually concerned with creation of geometrically accurate models from transparent silicone rubbers. However, the time of manufacture has been high – with lead time upwards of 2 weeks. Cost of manufacture has also been high – shown to be between 600 and 2000 EUR per model [16] – due to requiring two sets of molds, for outer and inner geometry, and material waste [17]. From early 2000s aneurysm models have been manufactured using rapid prototyping and 3D printing (referred to jointly from now on as ‘3D printing’) [9, 10, 18–22]. A CAD model is prepared in the computer which then instructs the 3D printer. The ideal is a one-step process in which the final model is produced directly from 3D printing. In some cases a 2- or 3-step process may be required involving manufacture of a mold from the solid 3D printed core. Low-cost AAA phantoms have been accurately 3D printed using opaque and rigid thermoplastics such as polylactic acid (PLA) [18, 23]. 3D printed accurate AAA models have also been achieved using flexible, transparent material although these have involved the use of expensive 3D printers such as Stratasys printers (typically above £70,000 for their printers) with Polyjet technology [20]. This high cost leads to a restriction in widespread clinical application in areas such as surgical training or other repetitive processes.

The main aim of this study was to investigate the possibility of producing an accurate, flexible, semi-transparent patient-specific AAA model using a low-cost FDM machine. A secondary goal was to determine what parameters exert the most influence on geometrical accuracy and model transparency, and from this establish the optimum settings to allow faithful replication of complex patient geometry.

## Methods

### Materials

Thermoplastic polyurethane (TPU) filament, manufactured by Ninjatek, called Ninjatek Cheetah Water (USA), and polyvinyl alcohol (PVA) filament, manufactured by Ultimaker, were purchased from Create Education Limited, UK. Material properties for Ninjatek Cheetah can be seen in Additional file 1: Table S1 (Appendix).

### 3D printer

The Ultimaker 3 FDM 3D printer was used (Create Education Limited, UK). It has the capability for a minimum thickness layer of 20 μm, dual extrusion to utilize a second extruder for water-soluble support structure printing, and automatic build-plate levelling. See the technical specifications of the used Ultimaker 3 3D printer in Additional file 1: Table S2 (Appendix).

### G-code and 3D printing of AAA

The patient-specific aneurysm modelled in this study was reconstructed from the computed tomography (CT) scan data of a patient undergoing AAA surveillance as part of the MA^3^RS clinical trial (http://www.isrctn.com/ISRCTN76413758) [3]. CT scanning of the aorta was performed from just below the thoracic arch to below the iliac bifurcation (Aquilion One, Toshiba Medical Systems Ltd., UK). The slice thickness was 0.5 mm, with a pixel size of 0.625 mm.

**Fig. 1:**
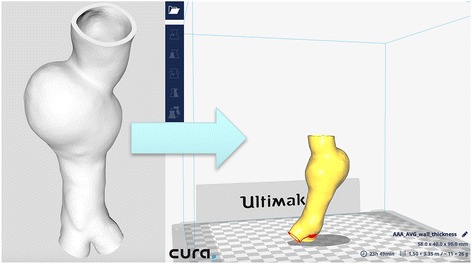
Picture of the 3D reconstruction from the patient’s AAA geometry. The picture was obtained from CT scan data, which was transferred to the software Cura (version 2.5, Ultimaker, Geldermalsen, Netherlands) for layer slicing and setting the 3D printing parameters

The process for segmentation and reconstruction of the patient-specific AAA CT scan data has been described in detail previously [24]. Briefly, segmentation and reconstruction were carried out in commercial software (Mimics innovation suite, Materialise, Belgium) using manual and semi-automatic thresholding tools, volume preserving smoothing (to remove scanning artefacts) and meshing operations were then performed in 3-matic (Materialise). Finally, 3D meshes were exported in the printer compatible computer aided design (CAD) STL format. Figure 1 shows the computerized 3D geometry of the used AAA with an average wall thickness of 2 mm. The chosen STL file was repaired to remove potential gaps in the geometry and to ensure smooth surfacing before 3D printing. The STL file was then transferred to the software Meshmixer (Autodesk Inc., San Rafael, CA, USA) for mesh repair and element normalization. The updated STL file was then transferred to the slicing software Cura (version 2.5, Ultimaker, Geldermalsen, Netherlands) for 3D printing (Fig. 1). This software was used to automatically slice the file into suitable layers for 3D printing with the Ultimaker 3 3D printer. Print bed temperature was set as 40 °C in Cura 2.5 to ensure material adhesion to the print bed, while not exceeding the temperature limit where warping occurs (a phenomenon where the geometry closest to the print bed distorts due to an excessive print bed temperature being used). PVA support was required for the overhanging geometry of the AAA and so the printer’s second extruder was primed for support structures with a brimmed adhesion layer between the model and the build plate for ease of removal with a spatula. The PVA printing parameters were fixed to the default recommended settings given in Cura 2.5 and are listed in Additional file 1: Table S3.

### Optimization of 3D printing parameters

The parameters layer height, wall thickness, infill density, print speed and print temperature were investigated for the best model quality, optimizing for transparency and flexibility. The optimized printing parameters were determined through benchmark tests on idealized geometry. Rings of 17 mm inner diameter with 2 mm wall thickness and 5 mm height were 3D printed (see Additional file 1: Figure S3) while altering the above-mentioned parameters. The diameter and wall thickness were chosen in accordance with similar cross-sectional geometry to the smallest section of the AAA model to ensure fastest printing time for efficient testing.

Each ring was printed twice with the same parameters to take reproducibility into account. For each data entry, the printed ring’s wall thickness was measured at six equidistant points with Vernier caliper, and averaged to give a comparative value with the 2 mm thickness set in the CAD model.

The results were taken in the following order: the effect of printing speed, infill percentage and printing temperature. While a chosen parameter was altered, the other parameters were held at the lowest value in their respective testing range.

### Measurement of average wall thickness

The printed AAA model was placed in a warm water bath for 3 h to allow the PVA support structure to dissolve. The model was then washed with warm water to remove excess PVA from the inside of the AAA and then cut into sections using scissors for 7 section measurements. These sections were measured at six equidistant points with Vernier callipers to give an accurate representation of average wall thickness for each cross-section. This overall average wall thickness could then be compared to the CAD file dimensions and other papers to give an idea of comparative manufacturing accuracy.

### Tear resistance testing

As discussed in Corbett et al. [14] and shown in Additional file 1: Table S4, silicone rubbers are good for AAA physical modelling due to high transparency and flexibility but struggle with barbed and hooked stent graft placement simulation due to low tear resistance.

TPU, like Ninjatek Cheetah, is a copolymer composed of both low and high polarity chain segments. The polar segments have a strong affinity with each other, creating strong polar bonding, giving stiffness and cohesion to the system. The non-polar chain segments allow the polymer to be flexible and induces good elongation properties. Since tear testing includes investigating the stiffness, maximum strength and strain at break, TPU looks like an ideal candidate.

**Fig. 2:**
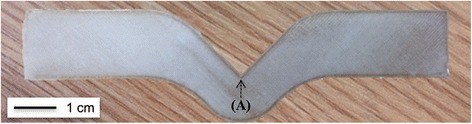
3D printed Ninjatek Cheetah Water TPU angle test piece that complies with ISO 34–1:2015 standards. This specimen was used for tear resistance test measurements with a 1 mm nick at point A

To investigate the use of Ninjatek Cheetah for this application, tear resistance testing was conducted on the 3D printed samples in accordance with ISO standard 34–1:2015 using nicked angle test specimens (Method B). The procedure of this method requires a specific shape, which was 3D printed using the Ninjatek Cheetah Water TPU filament material (Fig. 2), with a 1 mm nick taken at point (a) using a knife to induce consistent tear initiation. The 3D printing parameters for the angle test piece manufacture can be found in Additional file 1: Table S5.

The specimens were set up to have the force applied vertically by an Instron 3369 tensile testing machine with a 1 kN load cell (Additional file 1: Figure S1 (Appendix)), at a crosshead displacement speed of 500 mm/min. The laboratory temperature was 21 °C, and the samples had been conditioned for 24 h in the laboratory at this temperature before the test took place.

The details on the test piece geometry can be seen in Fig. S2. The mean thickness of the TPU angle test piece is 1.972 ± 0.007 mm (see Additional file 1: Table S6).

## Results

### Effect of printing speed

**Fig. 3:**
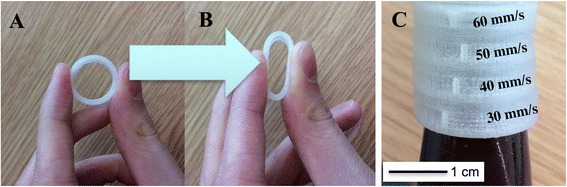
3D printed rings with 2 mm wall thickness for the application of AAA modelling. **a** and **b** The 3D printed rings are flexible. **c** Rings printed with 30, 40, 50 and 60 mm/s printing speeds (see Additional file 1: Table S7 in the Appendix for the rest of the printing parameters) had fair transparency and a noticeable join line that was constant throughout all prints

The effect of increasing printing speed was investigated for relationships with transparency and accuracy. For printing speeds between 30 mm/s and 90 mm/s, it was seen that the overall transparency of the material remained constant although the result was poor with only moderate transparency. In each case, the rings were flexible (as shown in Fig. 3a and b) but also had a noticeable join line, seen in Fig. 3c. Meanwhile, the 80 mm/s and 90 mm/s rings had flawed wall geometry with material stringing and gaps near the bottom of the print.

Precision thickness data can be seen in Table 1 (see details in Additional file 1: Table S8 in Appendix). The results show that higher printing speed results in increased difference from the CAD model.

**Table 1:** Wall thickness of rings with increased printing speed

Print speed [mm/s]	Average wall thickness [mm]	Difference from CAD model [%]
30	2.00	0.0
40	2.02	1.0
50	2.05	2.5
60	2.07	3.5
70	2.08	4.0
80	2.10	4.9
90	2.17	8.5

### Effect of infill percentage

**Fig. 4:**
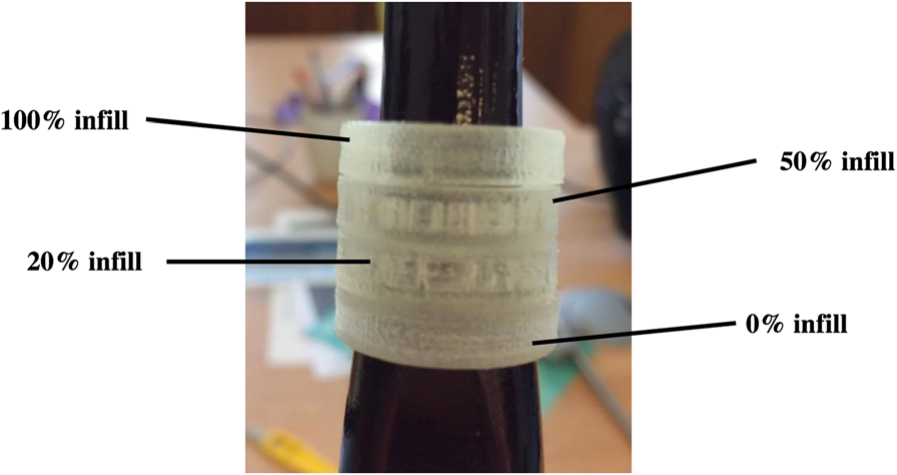
The infill density changes the sample transparency. The 100% infill density displays better transparency than the 0% infill density. The 20 and 50% infill densities have partial layer fill, clouding the transparency. With 100% infill the sample is has no air gaps; thus, no clouding of optical properties appears

The infill density effect was studied for the transparency of the samples. The infill percentage governs the quantity of plastic deposited to form the internal structure of the model; in this case, the arterial wall. Four different infill percentages of 3D printed ring samples were tested for transparency: 0% (no infill), 20% (light infill), 50% (dense infill) and 100% (solid). The 0% and 100% infill rings had even distribution of layers with the 100% infill providing greater transparency than the 0% infill (Fig. 4). While transparency seemed to increase with infill percentage, the 20% and 50% infill rings had lines of material inside due to the partial filling of the layers that clouded areas of transparency.

The accuracy of the printed model with the 0% infill was very high, as the mean wall thickness was 2.003 ± 0.015 (see Additional file 1: Table S9 in Appendix). However, the accuracy of the sample decreased with increased infill percentage. 100% infill density results in a significant, 2.9% increase in the wall thickness of the model (Table 2). The thickness and accuracy of the 3D printed rings with the four different infill percentages is displayed in Table 2.

**Table 2:** Wall thickness of rings with increased infill percentage

Infill percentage [%]	Average wall thickness [mm]	Difference from CAD model [%]
0	2.00	0.0
20	2.03	1.5
50	2.03	1.5
100	2.06	2.9

### Effect of printing nozzle temperature

**Fig. 5:**
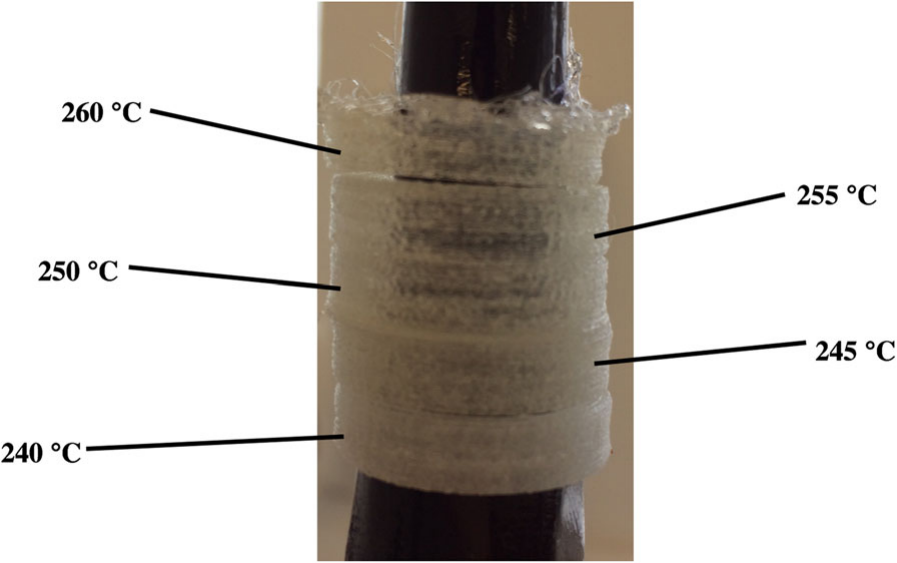
3D printing temperature effects on the transparency. With increasing printing nozzle temperature the transparency increases

Five different printing temperatures were investigated for effect on temperature and transparency: 240, 245, 250, 255 and 260 °C. The printed rings show visually that the transparency increased with increased temperature (Fig. 5). This is most likely due to the better fusion of the printed filaments in the material. The 3D printed rings became more rigid and rough as the temperature was increased. At a temperature of 260 °C, the print failed due to bubbles in the melted filament due to excessive temperature.

The precision thickness data of the printed samples can be seen in Table 3 (see details in Additional file 1: Table S10 Appendix). The results show that printing at 255 °C produces the lowest deviation from the wall diameter defined in the CAD model (2.01 vs 2.00 mm).

**Table 3:** Wall thickness of rings with increased printing temperature

Print temperature [°C]	Average wall thickness [mm]	Difference from CAD model [%]
240	2.09	4.5
245	2.09	4.5
250	2.06	3.0
255	2.01	0.5
260	1.94	3.0

### Optimized parameters

The optimization tests for the parameters printing speed, printing nozzle temperature and infill percentage, showed that the best parameters for 3D printing accurate and transparent AAA using the Ninjatek Cheetah Water TPU filament were 30 mm/s printing nozzle speed, 255 °C printing nozzle temperature, and 100% infill (Table 4). These parameters are significantly different from the printing guidelines provided by Ninjatek for the Cheetah Water filament (see Appendix, Table S11 and Table S12 of Appendix).

**Table 4:** Optimized 3D printing parameters for AAA modelling

Printing speed	30 mm/s
Printing nozzle temperature	255 °C
Infill percentage	100%

### 3D printed AAA model

**Fig. 6:**
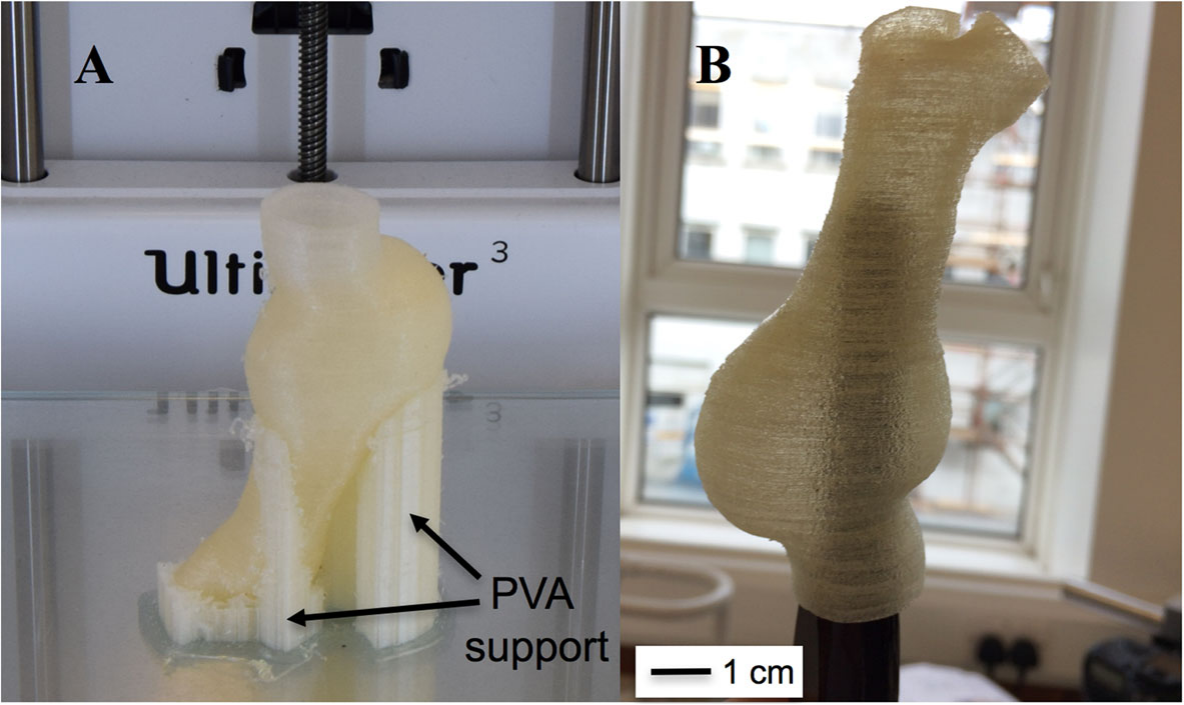
**a** AAA model printed with PVA support structure. **b** model after PVA support was dissolved in warm water bath. A black object was placed inside to display level of transparency

The patient-specific flexible semi-transparent AAA model was printed in 25 h and 3 min. This method used 20 g (2.63 m of filament) of Ninjatek Cheetah and 26 g (3.35 m of filament) of PVA. Considering the price of the used filament, the cost of an AAA model is around £4.50. The printed AAA can be seen with and without the PVA support structure in Fig. 6. The transparency of the 3D printed AAA model seems to be sufficient for surgeons to inspect the position of an instrument placed inside the structure.

**Fig. 7:**
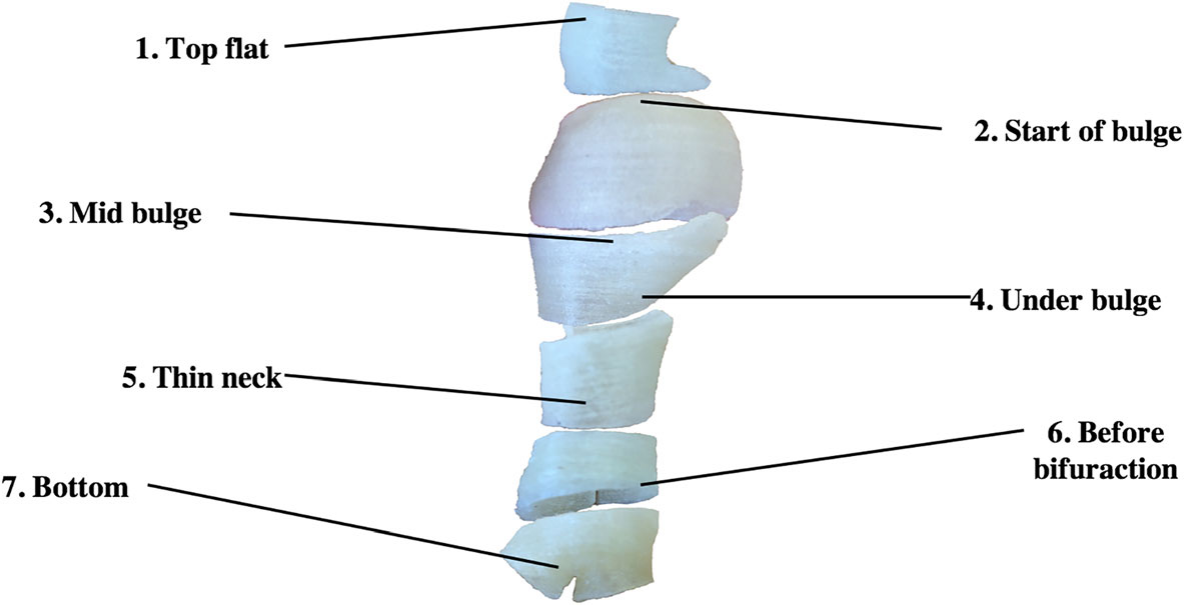
Printed AAA model with cut sections for use in accuracy testing

The accuracy of the printed AAA was calculated by performing measurements of the AAA cross-section wall thicknesses at 7 different sections: top flat, start of bulge, mid bulge, under bulge, thin neck, before bifurcation and bottom (see Fig. 7 and Table 5).

**Table 5:** Measured cross-section wall thicknesses of printed AAA model

Section name	Average wall thickness [mm]	Difference from CAD model [%]
Top flat	2.07	3.5
Start of bulge	2.14	7.0
Mid bulge	2.03	1.5
Under bulge	2.01	0.5
Thin neck	2.03	1.5
Before bifurcation	2.04	2.0
Bottom	2.06	3.0

Six equidistant cross-section wall thicknesses were measured for each section, and the averaged wall thickness was calculated to be 2.05 mm, which is just 2.5% higher than the wall thickness in the CAD model (see details in Additional file 1: Table S13 in Appendix). Thus, the accuracy of the 3D printed AAA is very close to the one specified in the CAD model.

### Tear resistance test results

**Fig. 8:**
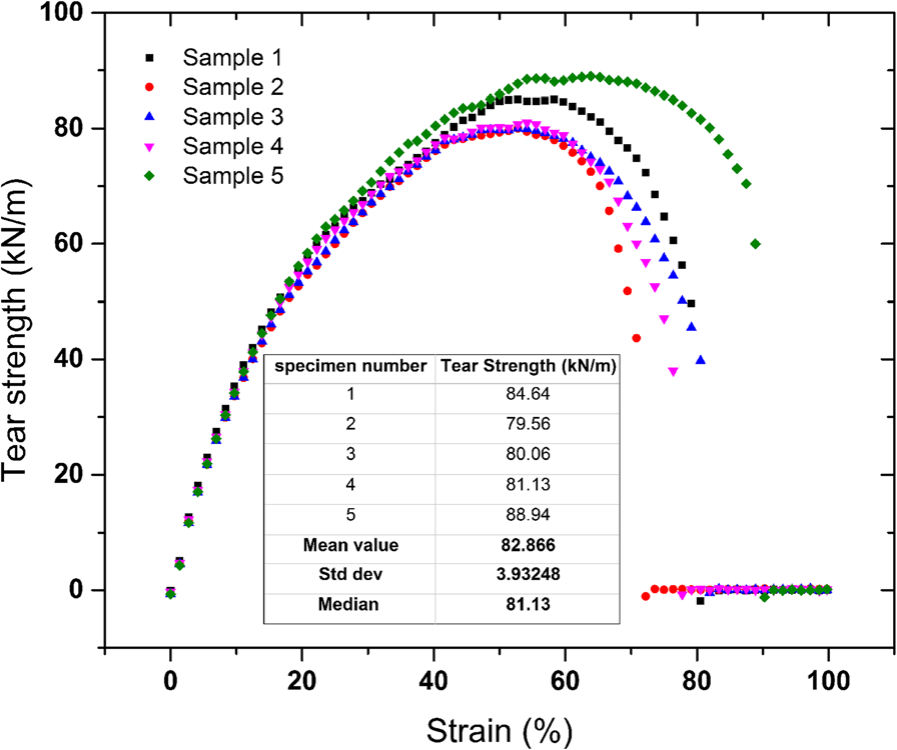
Ninjatek Cheetah tear strength tests for nicked angle test specimens according ISO standard 34–1:2015

The five samples exhibited a similar initial mechanical behavior, and the maximum tear strength gave a mean value of 82.866 kN/m, with a standard deviation of 3.932 kN/m, which is 4.74% deviation from the mean value (see Fig. 8). These results highlight the reproducibility and reliability of the 3D printing processing for these flexible samples. Silicone rubber tear strength is usually between 1 and 20 kN/m at room temperature [25–28]. Thus, the 3D printed Ninjatek Cheetah material displays significantly better tear strength properties than silicone rubbers.

## Discussion

This study aimed to produce a flexible and robust physical model of a patient-specific AAA to aid endovascular aneurysm repair education and planning in a time and cost-effective manner. The FDM 3D printing technique is a relatively fast and inexpensive way to manufacture an AAA model for a particular patient. Since CT scanning typically takes approximately 15 min [29], the total processing and manufacturing procedure could feasibly be done in less than 48 h. This is a significantly reduced time compared with that for lost wax casting procedures using CNC machined molds and other rapid prototyping procedures that require at least 2 weeks to manufacture a model [30].

The physical model was optimized by investigating the effect of altering printing parameters, and this showed that an increase in printing nozzle temperature increased the transparency of the Ninjatek Cheetah TPU material. At a temperature of 260 °C, bubbles began to form during printing due to excessive temperature, and this phenomenon created defects in the geometry. An infill percentage of 100% was associated with an increase in transparency, most likely as light only needed to travel through one solid wall of material (with the 100% infill model) compared to two walls (0% model) or several walls (20% and 50% models), all of which would attenuate transmitted light more. Despite this optimization for transparency of the Ninjatek Cheetah material, the result provided only fair transparency. A noticeable characteristic of all the printed models was that horizontal transparent layers tended to lie between layers of opaque material, where the nozzle had deposited the melted filament. A fix for this would be to use a larger layer thickness, however this would be at the expense of geometrical accuracy. Another possible way to increase transparency could be decreasing the cooling temperature ramp, or use a solvent vapor to partially dissolve the 3D printed sample.

While printing temperature and infill percentage only altered the geometry of the model compared to the CAD model by a range of around 2%, increasing printing speed had a more adverse effect on accuracy. Although an increase in dimensional error of around 8% was shown when increasing print speed from 30 to 90 mm/s, the printing time decreased to 14 h and 11 min for printing the AAA model. However, when comparing the average wall thickness error of approximately 8.5% for a final AAA model manufactured by 3D printing at 90 mm/s with a 14% error in geometry using casting techniques such as that of Doyle et al. [13], it appears that using higher printing speeds would be acceptable if print speed was a greater priority than exact geometry.

In terms of varying accuracy with section location, the most inaccurate area of the AAA model was at the top of the bulge of the aneurysm (7% error). Noticeable characteristics of this area are the complex curved geometry and the lack of PVA support inside or outside the model. It was a common trait across all measurements taken for the thicknesses to exceed that of the CAD model. This suggests that the material expands when deposited, and therefore it can be assumed that surrounding the material with a PVA support (which is always printed before the primary material when building each layer) must reduce material expansion. This expansion would also explain why the only other section to have PVA support absent both inside and outside (i.e., the very top of the model) has the second largest error (3.5%) despite having relatively simple geometry compared to other sections. To further support this argument, the under-bulge section was the most precise area of the model (0.5% error) and was almost completely surrounded by PVA support (see Fig. 6a). Nevertheless, the 2.5% error between the average thickness of the AAA physical model and that of the CAD model is comparable with the precision of dimensions measured in other 3D printing AAA studies and can therefore be deemed acceptable [19].

The 3D printed AAA model has good tear resistance to sustain performance for multiple repetitions of endovascular aneurysm repair testing. Silicone rubbers used for modelling AAAs have low tear resistances [25–28], which could cause problems when placing stent grafts inside the model, particularly for hooked or barbed stents, or for procedures with multiple repetitions on the same model. From the testing carried out in this study, it was found that the average tear resistance of Ninjatek Cheetah TPU is ~13–40 times higher than the tear resistance of commonly used silicone rubbers, like Sylgard or PDMS (see Additional file 1: Table S4 in Appendix for comparison). Thus, the tear resistance of Ninjatek Cheetah exceeds silicone rubbers while still maintaining good levels of flexibility with a Young’s modulus of 26 MPa, as shown in Additional file 1: Table S1 (Appendix). However, it should be also considered that directionality is important for 3D printed materials as they are not isotropic. Differences in printed filament orientation will likely lead to different material properties and may affect the value of tear strength obtained.

The 3D printed AAA model had lower cost (4.50 GBP per model), shorter manufacturing time (25 h 3 min) and higher accuracy (~2.5% error) compared to other methods reported in literature [13, 30]. The cost per model was in a similar range to opaque, rigid 3D printed AAA model (3.00 EUR per model) reported by Bangeas et al. [23]. Barriers to more widespread clinical adoption of 3D printing are the high costs associated with the high-end state of the art 3D printing systems e.g. Stratsys Objet or Connex series (typically above £70,000 for their printers) are diminished, as the Ultimaker 3 printer costs approximately £3300.

Although the 3D printer achieved an AAA model, this study is still not without its limitations. The final AAA model was only semi-transparent, and had poor transparency compared to silicone rubbers with optically-clear transparency. Although objects placed inside the model (see Fig. 6b) could still have their positions identified easily and effectively; a stent graft would have to be placed inside the model to give a fair evaluation of effectiveness. Investigations into using alternative materials which have higher transparency and with material with higher compliance, would improve the model drastically. Additionally, biocompatible studies on the material used to make the model are also required if this model is brought into the clinic as a surgical aid when planning and surgery are taking place.

The STL files accessed for this study lacked geometrical data for the iliac arteries. Further study on examples with more complex geometries should be conducted, such as AAAs that extend into the iliac arteries or those with short neck distance to the renal arteries, as most AAAs are not infrarenal. Furthermore, next to the wall thickness other metrics should be analyzed as well, such as inner diameter and location of branch vessels to give a more comprehensive evaluation of model accuracy. With regards to model accuracy evaluation, the final wall thickness measurements were compared to the STL file and not the CT scan geometry. Therefore, there could be geometrical errors associated with CAD file processing, such as errors from segmentation. As for the wall thickness measurements, using scissors to cut the model into sections may have influenced the measurement accuracy in case the sample was not cut in the axial plane. In the current study this effect is below the measurement threshold (<0.01 mm).

Overall, with the levels of transparency, flexibility and tear resistance provided by the 3D printed part, the patient-specific AAA model could potentially be used for surgical planning for individual patients as well as for surgeons practicing endovascular aneurysm repair stent graft. Different types of stent grafts exist such as passively fixating, hooked and barbed, and stents that utilize bifurcation to restrict migration. Models could be 3D printed for each of these scenarios for practice in identification, and to familiarize doctors with AAAs that could be difficult to visualize with only a 3D computer image. Conversely, due to lack of similarity to aortic properties, the 3D printed AAA model would not be useful for endovascular aneurysm repair simulation, peak wall stress or fluid flow testing.

## Conclusions

This paper investigated fused deposition modelling (FDM) 3D printing method for manufacturing abdominal aortic aneurysm (AAA) physical models for rupture prediction and prevention. The Ultimaker 3 3D printer was used for rapid prototyping of a patient-specific AAA using a semi-transparent thermoplastic polyurethane filament together with a PVA filament, for water-soluble support structure generation. Experimentation with printing parameters found that an increase in printing nozzle temperature to 255 °C and an infill percentage of 100% increased the transparency while maintaining precision. Increasing printing speed was found to have a detrimental effect on precision, with a 90 mm/s speed yielding an 8.5% discrepancy between CAD and physical model wall thickness compared to an error lower than 0.01 mm when using a 30 mm/s printing speed.

The final model had excellent accuracy (average wall thickness error of 2.5%), fair transparency (position of inserts could easily be discerned); good flexibility and high tear strength (found to be 83kN). This tear strength offers a particular advantage in allowing the placement of hooked or barbed stents inside the model without distorting the wall geometry.

Optimization of printing parameters allows for a geometrically accurate 3D patient-specific AAA model to be produced rapidly (in 25 h and 3 min) and at low cost (£4.50 per model) using a desktop FDM machine. While these FDM systems are unable to fully replicate the physical behaviors and mechanical properties required to replicate AAA analogues appropriate for conducting stress analyses or flow tests, once carefully optimized, they provide an efficient and highly accessible platform from which flexible and geometrically accurate AAA models can be produced. The 3D printed models resulting from this approach could be used for endovascular aneurysm repair education and planning.

## Additional files

Additional file 1: Appendix. (DOCX 445 kb)

Additional file 2: Video of AAA 3D printing summary. (MP4 14097 kb)

## Abbreviations

3D: Three dimensional
AAA: Abdominal aortic aneurysm
ABS: Acrylonitrile-butadiene-styrene
CAD: Computer aided design
CNC: Computer numerical control
CT: Computed tomography
FDM: Fused deposition modelling
FEA: Finite element analysis
PLA: Polylactic acid
PVA: Polyvinyl alcohol
STL: Standard tessellation language
TPU: Thermoplastic polyurethane
