# Supplementary material for: On the optimization of low-cost FDM 3D printers for accurate replication of patient-specific abdominal aortic aneurysm geometry
Source: 3D Print Med. 2018 Jan 17;4:2. doi: 10.1186/s41205-017-0023-2 (PMC5954792; doi:10.1186/s41205-017-0023-2)
Supplement: Supplementary file 1 — Appendix. (DOCX 445 kb) [file 41205_2017_23_MOESM1_ESM.docx]

**Appendix**

**3D printing for generating a patient-specific model of the abdominal aortic aneurysm**

1. **3D printer and material specifications**

**Table S1**. Ninjatek Cheetah material data from Ninjatek datasheet [1].

| **General properties** | **Imperial** | **Metric** |
| --- | --- | --- |
| Specific gravity | 1.22 g/cc | 1.22 g/cc |
| Moisture absorption – 24 hours | 0.18% | 0.18% |
| **Mechanical properties** |  |  |
| Tensile strength, yield | 1250 psi | 9 MPa |
| Tensile strength, ultimate | 5650 psi | 39 MPa |
| Tensile modulus | 3800 psi | 26 MPa |
| Elongation at yield | 55% | 55% |
| Elongation at break | 580% | 580% |
| Toughness | 17000 in*lbF/in^3^ | 117.2 m*N/m^3^ x 10^6^ |
| Hardness | 95 Shore A | 95 Shore A |
| Impact strength | 9.1 ft*lbf/in^2^ | 19.1 kJ/m^2^ |
| Abrasion resistance | 0.06 g | 0.06g |
| **Thermal properties** |  |  |
| Melting point | 428°F | 220°C |
| Glass transition | -11°F | -24°C |
| Heat deflection temperature at 10.75 psi/0.07 MPa | 165°F | 74°C |
| Heat deflection temperature at 66 psi/0.45 MPa | 120°F | 49°C |

The technical specifications (Table S2) of the Ultimaker 3:

**Table S2**. Ultimaker 3 technical specifications from the Ultimaker 3 user’s manual [2].

| **Ultimaker 3** | |
| --- | --- |
| Precision | 20-200 micron |
| Minimum layer thickness | 20 micron |
| Minimum wall thickness | 0.4mm (with 0.4mm nozzle) |
| Build size | 215mm x 215mm x 200mm |
| Filament diameter | 2.85mm |
| Print head travel speed | 30-300mm/s |
| Nozzle temperature | 180-280°C |
| Build plate temperature | 20-100°C |

**Table S3.** PVA support structure printing settings [3].

| Layer height | 0.06mm |
| --- | --- |
| Wall thickness | 1.0mm |
| Infill density | 0% |
| Printing temperature | 215°C |
| Top and bottom layer printing speeds | 20 mm/s |
| Infill printing speed | 35 mm/s |
| Brim adhesion width | 3 mm |

1. **Tear resistance comparison of Ninjatek Cheetah with silicone rubbers**

**Table S4.** Comparing tear resistance of Ninjatek Cheetah TPU with silicone rubbers

| **Material** | **Cross-linker/ Reinforcement filler** | **Tear resistance**  **(kN/m)** | **Hardness**  **(Shore A)** |
| --- | --- | --- | --- |
| Ninjatek Cheetah TPU | - | 83 | 95 |
| Sylgard 160 [4] | - | 2.1 | 56 |
| Sylgard 170 [5] | - | 3.5 | 41 |
| Sylgard 184 [6] | - | 2.6 | 50 |
| PDMS [7] | Silastic LSR 9280-40 | 37.7 | 40 |
| PDMS [7] | One-part RTV acetoxy cure, Dow Corning 732 | 4.9 | 25 |
| PDMS [7] | One-part RTV alcohol cure, Dow Corning1 737 | 6.48 | 37 |
| PDMS [7] | One-part RTV oxime cure, Dow Corning 739 | 5.6 | 35 |

(1) Data taken from Dow Corning official datasheets for relevant materials; (2) Data taken from Kuo (1999)

1. **Tear resistance testing procedure**

From the ISO standard 34-1:2015 entitled ‘Rubber, vulcanized or thermoplastic — Determination of tear strength’ [8], the procedure is described as:

- 1. **Procedure**

1. Measure the thickness of the test piece in the region in which tearing is expected to occur and in accordance with ISO 23529.
2. No measurement on any one test piece shall deviate by more than 2% from the median value of the thickness of that test piece.
3. If groups of test pieces are being compared, the median thickness of each group shall be within 7.5 % of the grand median thickness of all the groups.
4. Immediately mount the test piece in the testing machine.
5. Extend the test piece at a rate of separation of the grips of 500mm/min ± 50 mm/min for angle and crescent type test pieces and 100mm/min ± 10mm/min for trouser test pieces until the test piece breaks.
6. Record the maximum force for crescent and angle test pieces.
7. When using trouser test pieces, make an autographic recording of the force throughout the tearing process.
8. The tear strength $T_{s}$, expressed in kilonewtons per metre of thickness, is given in Equation S-1 as:

$$T_{s}= \frac{F}{d} (S1)$$

where:

$F$ is the maximum force, in newtons, when using method B or C, and the median force, in newtons, calculated in accordance with ISO 6133, when using method A;

$d$ is the median thickness, in millimetres, of the test piece

Express the results to the nearest kilonewton per metre.


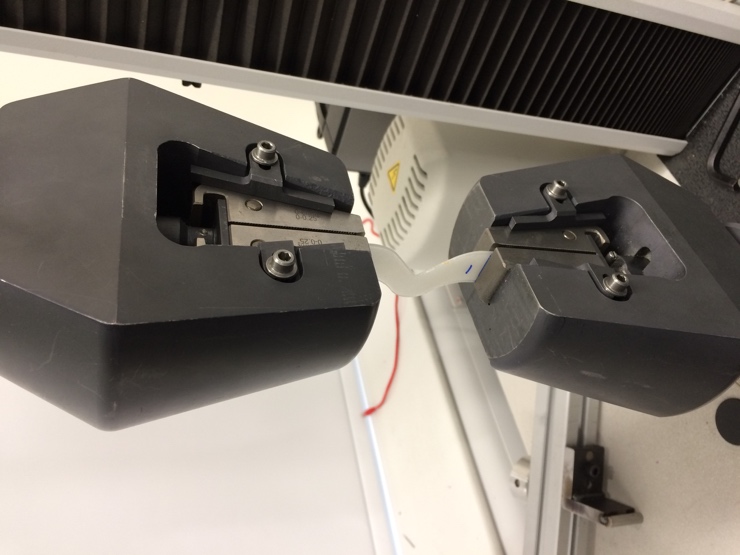

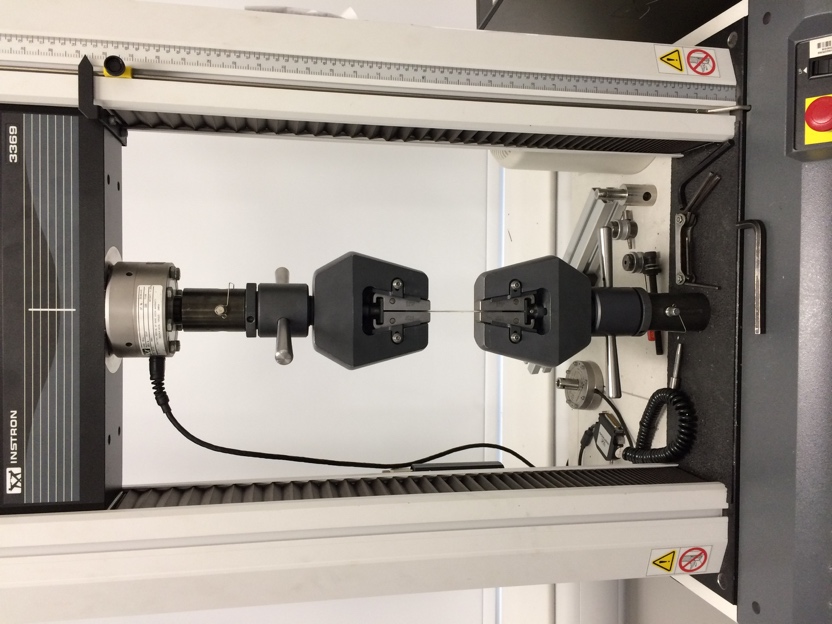


**Figure S1**. The test specimen had force applied by the testing machine in a vertical direction

1. **Sample details**

The sample chosen for tear resistance testing was the angle test piece show below in Figure S2.


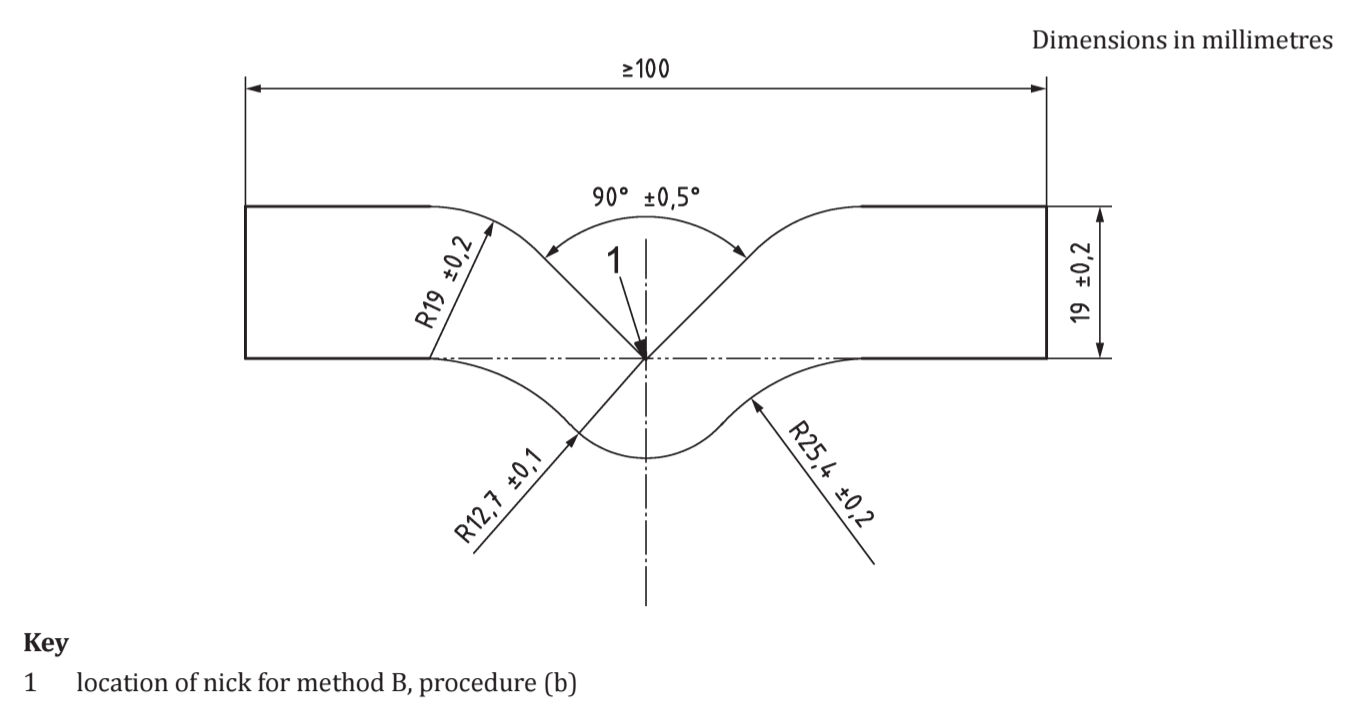


**Figure S2.** Angle test piece for tear resistance testing dimensions

The samples were manufactured by using the geometry defined in Figure S2 to generate a G-code for 3D printing from Ninjatek Cheetah thermoplastic polyurethane (TPU) filament. The 3D printer used was the Ultimaker 3 and had the following printing settings:

**Table S5.** Printing parameters for angle test piece manufacture

| **Printing speed** | 30mm/s |
| --- | --- |
| **Printing nozzle temperature** | 255°C |
| **Build plate temperature** | 40°C |
| **Layer thickness** | 0.06mm |
| **Wall thickness** | 0.4mm |
| **Infill percentage** | 100% |
| **Precision** | 20 microns |
| **Extrusion type** | Bowden |

Five test specimens were 3D printed and left 24 hours before nicking each sample at Point 1 (displayed in Figure S2) with a 1 mm cut.

The 3D printing method manufactured the test specimens using a cross-layer pattern to give even material grain strength in all directions.

Table S6 below shows the result of the median thickness measurements taken of the five test specimens:

**Table S6**. Median thickness measurements of angle test pieces

| **Specimen Number** | **Sample thickness (mm)** |
| --- | --- |
| 1 | 1.970 |
| 2 | 1.965 |
| 3 | 1.965 |
| 4 | 1.980 |
| 5 | 1.980 |
| Mean value: | 1.972±0.007 |

1. **Average wall thickness measurements**


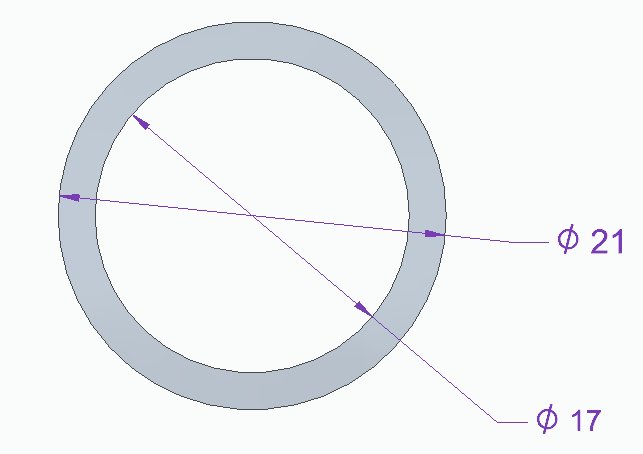


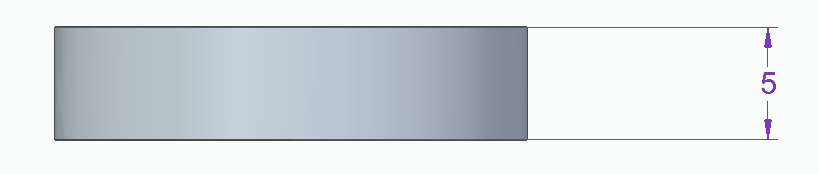


**Figure S3.** Ring test specimen dimensions used in precision and transparency optimisation for Ninjatek Cheetah TPU (dimensions in millimetres)

**AAA model wall thickness measurement full results**

**Table S8**. Wall thickness of rings with increased printing speed

| **Print speed**  **(mm/s)** | **Time for print**  **(mins)** | **Thickness measurement (mm)** | | | | **Average**  **(mm)** |
| --- | --- | --- | --- | --- | --- | --- |
|  |  | **1** | **2** | **3** | **4** |  |
| 30 | 23 | 1.98 | 2.01 | 2.02 | 2.00 | 2.00 |
| 40 | 18 | 2.02 | 2.00 | 2.04 | 2.02 | 2.02 |
| 50 | 16 | 2.04 | 2.03 | 2.09 | 2.05 | 2.05 |
| 60 | 14 | 2.08 | 2.05 | 2.07 | 2.06 | 2.07 |
| 70 | 13 | 2.08 | 2.06 | 2.11 | 2.05 | 2.08 |
| 80 | 13 | 2.10 | 2.08 | 2.07 | 2.14 | 2.10 |
| 90 | 13 | 2.15 | 2.14 | 2.17 | 2.23 | 2.17 |

**Table S9.** Wall thickness of rings with increased infill percentage

| **Infill Percentage**  **(%)** | **Thickness measurement (mm)** | | | | **Average**  **(mm)** |
| --- | --- | --- | --- | --- | --- |
|  | **1** | **2** | **3** | **4** |  |
| 0 | 1.98 | 2.01 | 2.02 | 2.00 | 2.00 |
| 20 | 2.04 | 2.02 | 2.04 | 2.00 | 2.03 |
| 50 | 2.03 | 2.05 | 2.03 | 2.01 | 2.03 |
| 100 | 2.05 | 2.06 | 2.05 | 2.07 | 2.06 |

**Table S10.** Wall thickness of rings with increased printing temperature

| **Print temperature**  **(°C)** | **Thickness measurement (mm)** | | | | **Average**  **(mm)** |
| --- | --- | --- | --- | --- | --- |
|  | **1** | **2** | **3** | **4** |  |
| 240 | 2.10 | 2.09 | 2.08 | 2.08 | 2.09 |
| 245 | 2.12 | 2.07 | 2.08 | 2.07 | 2.09 |
| 250 | 2.06 | 2.07 | 2.07 | 2.03 | 2.06 |
| 255 | 1.97 | 2.03 | 2.01 | 2.04 | 2.01 |
| 260 | 1.95 | 1.96 | 1.91 | 1.95 | 1.94 |

1. **3D printing pa****rameters for precision and transparency**

The printing guidelines provided by Ninjatek with the Cheetah Water filament are displayed in Table S6 below:

**Table S11.** Ninjatek Cheetah filament recommended printing guidelines

| Printing nozzle temperature | 230°C – 240°C (highest temperature for increased transparency) |
| --- | --- |
| Printing bed temperature | 20°C – 40°C (highest temperature for improved print bed adhesion) |
| Top and bottom layer print speeds | 30-45 mm/s |
| Infill print speed | 60-80 mm/s |

It should be noted that certain parameters were kept constant throughout the experiment. These are listed in Table S12 below:

**Table S12.** Experimental constant parameters

| Layer height | 0.06mm |
| --- | --- |
| Wall thickness | 0.4mm |
| Print bed temperature | 40°C |
| Retraction | Enabled |

The layer height, wall thickness and print bed temperature were all chosen in accordance with what would be used in printing of the AAA. The layer height and wall thickness were chosen as the minimum possible before under-extrusion occurs, where gaps are left in the print due to not enough material being deposited.

Once the parameters were chosen, the experiment followed the procedure listed below:

1. Set parameters to those displayed in Table S12
2. Set parameter to be altered at required value in range
3. Set all other parameters to lowest value in range
4. Import ring STL file with dimensions detailed in Figure S3
5. Export G-code to USB drive
6. Set Ultimaker 3 for automatic bed levelling for calibration
7. 3D print the ring
8. Retrieve printed model and investigate level of transparency and flexibility
9. Use Vernier callipers to measure 4 evenly spaced thicknesses around the ring
10. Repeat process, increasing the value of parameter in step 2

**Table S13.** Measured cross-section wall thicknesses of printed AAA model

| **Section name** | **Section No.** | **Cross-section thickness measurement (mm)** | | | | | | | **Average**  **(mm)** |
| --- | --- | --- | --- | --- | --- | --- | --- | --- | --- |
|  |  | **1** | **2** | **3** | **4** | | **5** | **6** |  |
| Top flat | 1 | 2.09 | 2.08 | 2.07 | 2.04 | 2.05 | | 2.08 | 2.07 |
| Start of bulge | 2 | 2.18 | 2.15 | 2.18 | 2.17 | 2.11 | | 2.04 | 2.14 |
| Mid bulge | 3 | 2.03 | 2.08 | 1.99 | 1.98 | 2.03 | | 2.06 | 2.03 |
| Under bulge | 4 | 2.01 | 2.00 | 2.04 | 2.00 | 2.01 | | 2.01 | 2.01 |
| Thin neck | 5 | 1.98 | 2.01 | 2.09 | 2.04 | 2.00 | | 2.03 | 2.03 |
| Before bifurcation | 6 | 2.07 | 1.98 | 2.11 | 1.99 | 2.01 | | 2.05 | 2.04 |
| Bottom | 7 | 2.05 | 2.08 | 2.04 | 2.07 | 2.06 | | 2.05 | 2.06 |
|  | | | | | | **TOTAL** | | | **2.05** |

**Tear resistance results**

Five test specimens were torn in total during this experiment using the angle test piece tear resistance test method B.


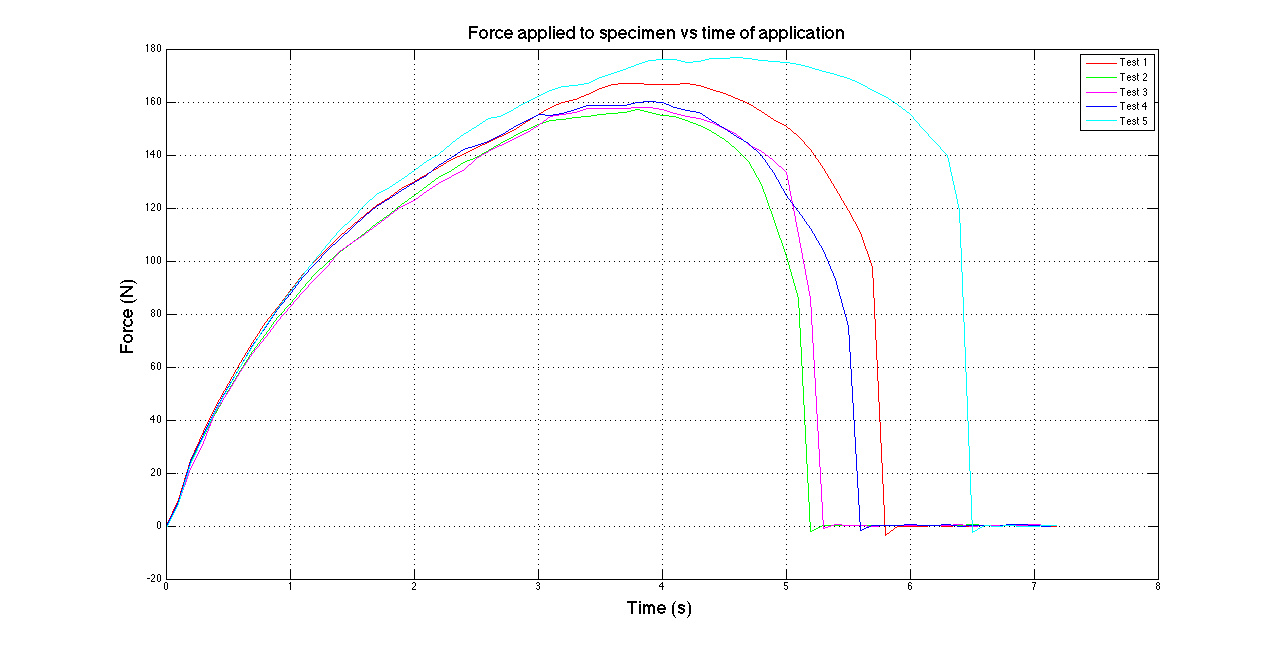


**Figure S4.** Variance of force with time with five test specimens for angle test piece tear resistance testing.

**Table S14.** Maximum force recorded for each test specimen

| **Specimen Number** | **Maximum force (kgf)** | **Maximum force (kN)** |
| --- | --- | --- |
| 1 | 17.03421 | 0.167 |
| 2 | 16.00339 | 0.157 |
| 3 | 16.10578 | 0.158 |
| 4 | 16.33883 | 0.160 |
| 5 | 18.0272 | 0.177 |

**References**

<https://ninjatek.com/wp-content/uploads/2016/05/Cheetah-TDS.pdf>

<https://ultimaker.com/download/61355/Ultimaker%203%20manual%20%28EN%29.pdf>

<https://ultimaker.com/en/resources/23003-how-to-print-with-ultimaker-pva>

1. <https://3.imimg.com/data3/AV/AB/MY-8890895/dow-corning.pdf>
2. <http://webservice.oxygenekatalog.dk/getdoc.aspx?docid=10493>
3. <http://webservice.oxygenekatalog.dk/getdoc.aspx?DocID=10494>
4. Kuo ACM. Poly (dimethylsiloxane). Polym. Data Handb. [Internet]. 1999;411–35. http://www.rubloffgroup.umd.edu/teaching/enma490fall03/resources/current/publications_etc/pdh-735(pdms).pdf
5. ISO 34-1 Rubber, vulcanized or thermoplastic — Determination of tear strength — Part 1: Trouser, angle and crescent test pieces. Int. Organ. Stand. 2010;1–18.
